# Missing Links in Middle School: Developing Use of Disciplinary Relatedness in Evaluating Internet Search Results

**DOI:** 10.1371/journal.pone.0067777

**Published:** 2013-06-26

**Authors:** Frank C. Keil, Jonathan F. Kominsky

**Affiliations:** Psychology Department, Yale University, New Haven, Connecticut, United States of America; ICREA-University of Barcelona, Spain

## Abstract

In the “digital native” generation, internet search engines are a commonly used source of information. However, adolescents may fail to recognize relevant search results when they are related in discipline to the search topic but lack other cues. Middle school students, high school students, and adults rated simulated search results for relevance to the search topic. The search results were designed to contrast deep discipline-based relationships with lexical similarity to the search topic. Results suggest that the ability to recognize disciplinary relatedness without supporting cues may continue to develop into high school. Despite frequent search engine usage, younger adolescents may require additional support to make the most of the information available to them.

## Introduction

In the last decade the use of internet search-engines has skyrocketed among American middle school students [1]. Indeed, it has been suggested that internet usage among children and adolescents has become so prevalent that there is now a “digital native” generation in which the internet has always been a central part of that generation's lives [2]. Although internet usage can describe a wide variety of activities, information-seeking is one of the most ubiquitous uses of the internet among middle school and high school students that use the internet at least once a week [3]. Despite this heavy usage, this age group may have intriguing deficits in the ability to discern relevant information among internet search results.

Children certainly perform worse than adults on internet search tasks (e.g., [4]) and they do so in ways that are correlated with other facets of cognitive development. For example, more advanced epistemological beliefs predict more successful selection of search terms [5]. However, while choosing the right search terms is important for successfully finding information on the internet, it is equally important to be able to identify the relevant items in the results of those searches.

Even adults may have trouble identifying the most relevant results returned by a search engine. For example, adults tended to rely on the top search results provided by Google™, even when they were less relevant than items ranked lower on the page [6]. The assessment of relevance may be even more difficult for younger internet users. Younger search users tend to have problems judging the relevance of results, particularly due to over-reliance on surface features ([7], [8], for review). For example, middle school students (ages 10–13) may be led astray by the capitalization of keywords or by their limited reading comprehension skills [7]. They may also be seduced by surface lexical matches (e.g., in searching for an answer to the question “Why do people catch colds?”, they might be attracted to a top ranked item entitled “Cold weather may pose health risks”). These surface matches, however, may not be the most persistent source of errors. In fact simple lexical matches may be relatively easy to learn to override, through strategies such as asking if the two uses of the word have the same meaning. Rather, even adolescents may overlook important results that require the ability to cluster knowledge solely by discipline.

People do not construe knowledge as being distributed evenly throughout the world. Rather, people expect knowledge to be clustered in predictable ways. One principle by which we navigate knowledge in the world is discipline-based clustering: organizing knowledge based on underlying causal principles. For example, advanced knowledge of how a bicycle stays upright should suggest similar understanding of how a spinning top works, since they are both reliant on the fundamental causal discipline of physical mechanics. A deep understanding of the privileged nature of discipline-based knowledge clusters in most knowledge-based tasks does not typically emerge till at least the fourth grade (9–10 years old) [9]. Even sixth graders (11–12 years old) have difficulty discerning discipline-based expertise when it is pitted against other ways of clustering knowledge, such as by common goals or superficial topic matches [10]. Although preschoolers can sense discipline-based ways of clustering knowledge when presented without distracters [11], these preferences are fragile and difficult to maintain in the face of competing ways of sorting knowledge.

Search engine results may provide a real-world example of when this type of information competition might present adolescents with challenges. In addition to result titles and summaries that have lexical similarities to a search term but no relevance in discipline, some results may be highly relevant in discipline but lack lexical similarities in the title or summary paragraphs. This is made possible by the use of “metadata” [12], information made available to the search engine about the contents of the page that does not rely on what is presented to visitors of that page. For example, a recent Google™ search for “how does a music player work” in the fall of 2011 turned up a result titled “How MP3 files work” as the second result from the top. Neither the title nor the summary of this result included the phrase “music player.” However, it is clear to us as adults that this information belongs to the same discipline-based knowledge cluster. Because many modern music players are in fact MP3 players, knowing how an MP3 file stores music and is interpreted is highly relevant to how a music player works. The meta-data of the page that the result linked to included the phrase “MP3 player,” among others. That inclusion, in combination with other algorithms Google uses in evaluating search results, brought it (appropriately) near the top of the search results.

Adolescents may have difficulty recognizing this kind of search result as relevant information. In addition to the general difficulties with discipline-based knowledge clustering noted above, recent work has found that children require “images and titles related to the contents” in order to identify relevant information [13], building on the over-reliance on surface features found in previous work [8].

The current work seeks to better characterize what information middle school and high school students do and do not use in evaluating search engine results. We created artificial search engine results that were specifically designed to separate and test the role of lexical similarity and discipline-based relatedness in evaluating relevance. In addition, we tested a wide age range, from sixth graders (minimum age 11 years) to college students (∼18–20 years), to characterize any developmental changes in how these information types are used.

In this study we created four types of search results by manipulating two factors: relatedness in discipline and lexical similarity. It is important to note that these two can be manipulated independently, leading to a total of four item types, each with distinct predictions across the age groups we studied. When results match in both discipline and lexical similarity, they can be thought of as “hits,” i.e., the most informative results for a given search query. The opposite case is a result that has neither disciplinary relatedness nor lexical similarity, which could be called a “miss.” We expected that the contrast between a “hit” and a “miss” would be obvious to all age groups, though based on previous work on children's challenges in search engine use (e.g., [7]), some developmental improvement would not be greatly surprising.

Results that have either disciplinary relatedness or lexical similarity, but not both, are the key items in this study, and where we expect the greatest developmental effect. Adults should recognize that disciplinary relatedness is key to relevance while simple lexical similarity in the absence of disciplinary relatedness is not. In contrast, younger participants may not be able to recognize disciplinary relatedness as a cue to relevance, especially with lexical similarity as a competing cue. Even though members of our youngest age group were in sixth grade, the results of Danovitch & Keil (2004) suggest that they might still underrate the relevance of items that are related in discipline but lack lexical similarity. Based on previous work, we expected a significant increase in the ratings of discipline-related items in the high school years, but because we included a more complete spectrum of ages than previous work, we could not predict what specific ages would differ from each other.

## Methods

### Ethics statement

All methods and materials were approved by the Yale University Human Subjects Research Committee under the protocol title “The emerging awareness of the organization of knowledge” (ID# 07060028715).

### Participants

Adolescent participants were drawn from two public school districts in southern Connecticut. Students brought home a consent form, which their parents signed if they wished their child to participate, and an assent form students signed if they wished to participate. Our sample consisted of every student for whom we received a signed consent form and signed assent form. Adult participants were undergraduates recruited from the university's introductory psychology subject pool and the broader student population. SES demographics were comparable for all age groups. Adults provided written consent for their participation. All participants were informed that they were allowed to withdraw from the study without consequences.

Participants consisted of 52 sixth graders (28 male, 24 female, mean age 11.9 years), 36 seventh graders (25 male, 11 female mean age 12.8 years), 44 eighth graders (18 male, 25 female, 1 did not report, mean age 13.7 years), 36 ninth graders (20 male, 16 female, mean age 14.9 years), 27 tenth graders (8 male, 19 female, mean age 15.9 years), 31 eleventh graders (14 male, 17 female, mean age 16.9 years), 23 twelfth graders (13 male, 10 female, mean age 17.9 years), and 39 adults (14 male, 21 female, 4 did not report, ages were not recorded but all were college students over the age of 18).

### Materials

Thirteen simulated Google™ search result pages were created. Each page consisted of a query and ten simulated search results, visually identical to a printed page of search results from Google™ in font selection, color, size, and page arrangement. The search results did not represent what would actually come up for that search topic. Most were fabricated and did not represent real websites while a few were based on, but not identical to, results for real websites. Stimuli of this type have been used before in previous work investigating reading comprehension and types of surface cues in internet search results [7]. Four versions of the study packet were made from four random orders of presentation of both the search topics and the results for each topic. The topics came from several disciplines including economics, psychology, chemistry, engineering, and biology. These domains were chosen because they have often been used in studies of discipline-based knowledge (e.g., [10]). A list of all the search topics can be found in Table 1.

**Table 1:** List of all search topics.

Why do airplanes have differently shaped wingsWhy does sugar dissolve in water
Why does a flu vaccine work
Why do some people have violent tendencies
Why does soap clean the dirt off of clothes
Why does a rainbow always have the red stripe on top
Why are some people still best friends after fights
Why do bridges need to be coated with special paint
Why can bigger companies make cars cheaper
Why do some siblings have the same blood type
Why do we repeat phone numbers to remember them
Why does a roller coaster stay on the tracks
Why does your skin heal after it has been cut

NOTE: The order of the topics in this table is one of four random orders used in the experiment. The order of results within each topic was also randomized across these four versions.

The primary manipulation was whether search results were related to the topic in discipline (DIS), lexical similarity (LEX), both, or neither. For each set of ten results, one result matched in both discipline and lexical items with the search topic (HIT items), three matched in discipline but had none of the same words as the search topic (DIS items), five had words from the search topic but were not in the same discipline (LEX items), and one matched in neither discipline nor words (MISS items). Table 2 shows examples of each item type from the search topic “why do bridges need to be coated with special paint”.

**Table 2:** Example of each type of result for the search topic “why do bridges need to be coated with special paint”.

	“HIT” Result (+DIS/+LEX)	“DIS” Result (+DIS/-LEX)	“LEX” Result (-DIS/+LEX)	“MISS” Result (-DIS/-LEX)
Link titles	“Issues impacting bridge painting: an overview”	“Strong glue bonds can still be broken”	“NWGNA: Volunteer paint the bridges!”	“Monopoly.com – the official site of Monopoly by Parker Brothers”
Link summaries	Although immersion of a coating in salt water alone is a severe environment, periods of wetness and heat can affect the paint because the salt concentration…	Glue is a very beneficial adhesive to use, however some conditions may alter its efficiency. The composition of glue is made in such a way to resist…	Historic Bridge at Prevost by Rumblin We need volunteers to come help paint this beautiful bridge. If you would like some more info on when to meet…	The board game that offers the vicarious thrill of getting rich quick. Site includes tips and strategies, history, tournaments, eCards, Park Place Player's …

There were a total of thirteen topics (see Table 1) and ten results per topic, one HIT, one MISS, three DIS and five LEX.

### Procedure

Participants were told that they were evaluating a new search algorithm for Google™. They were asked to rate on their own how relevant each search result was to the search topic on that page on a scale ranging from 1–4, with 1 = “Highly irrelevant” and 4 = “Highly relevant.” Participants were instructed that, when evaluating relevance, they should consider how well the webpage that corresponded to each link would answer the question in the search topic. Participants were debriefed immediately after the study and told that the searches were simulated and their data was not part of a Google™ initiative. In middle schools, all participants at a given grade level were run in one thirty-minute session, and all high school grades were run in one thirty-minute session. An experimenter monitored all sessions and ensured that participants were not discussing or comparing their ratings during the experiment. Adults had no time restrictions and were run one at a time in the lab in a quiet, secluded room. Most participants took 20–25 minutes to complete the study.

### Analysis strategy

We planned to examine four dependent measures from the relevance ratings of the different item types, each one providing different insight into the developing ability to evaluate relevance. In order to examine developmental changes in the ability to recognize relevance at all, we examined a difference score for HIT and MISS items, hereafter referred to as HIT-MISS. This measure indicated how well participants could make use of both relevance cues together. In order to examine a shift toward discipline-based knowledge clusters, we also calculated a difference score for DIS and LEX items, hereafter referred to as DIS-LEX. This measure indicated the degree to which participants favored discipline-based clustering over mere lexical similarity in evaluating relevance.

We also examined the average ratings for DIS and LEX items independently. We expected that there would be a developmental shift towards favoring DIS items and disfavoring LEX items, but our difference measure alone does not allow us to test both of these hypotheses. A significant effect of age on the DIS-LEX score (which we expected would be an increase) could indicate that the relevance ratings of DIS items increased with age or that the relevance ratings of LEX items decreased with age, or both.

We expected age to affect at least three of these measures, but because previous studies have not sampled every age in the range studied here, we did not have specific predictions about the ages at which we should see significant shifts. While we had general expectations that we would see a significant change in the high school years, because we analyzed each grade as a separate group we were not able to plan our age comparisons ahead of time, and therefore used post-hoc tests to better determine when these developmental shifts occur. Because we were primarily interested in developmental effects and no previous work on these topics has found consistent effects of sex, we ignored sex in our analysis.

## Results

Some student participants did not finish due to time constraints, and some participants elected not to fill out certain pages. These participants' data were included provided they filled out more than one page. Two sixth graders, one seventh grader, three eighth graders, and five adults were omitted for filling out the form improperly. One twelfth grader asked to be removed from the study.

We averaged scores across all items, and analyzed four dependent measures by grade: HIT-MISS, DIS-LEX, and then DIS and LEX independently. HIT-MISS and DIS-LEX were both calculated by taking the average scores of the appropriate item type and simply subtracting one from the other (e.g., HIT-MISS was the rating of the “HIT” item minus the rating of the “MISS” item). Because each rating was between 1 and 4, these difference scores had a range of −3 (1–4) to +3 (4–1). We conducted separate one-way analyses of variance (ANOVAs) by grade for each dependent measure.

There was a significant effect of grade on HIT-MISS (*F*(7,281) = 6.421, *p*<.001). Post-hoc tests revealed differences between middle-school grades and older participants. However, given that the possible range of HIT-MISS was -3 to +3, even the lowest-scoring grade, sixth graders (*M* = 1.97, SD = .71), were relatively close to ceiling, and far above chance levels (one-sample *t*-test vs. 0, *t*(50) = 19.85, *p*<.001).

**Figure 1:**
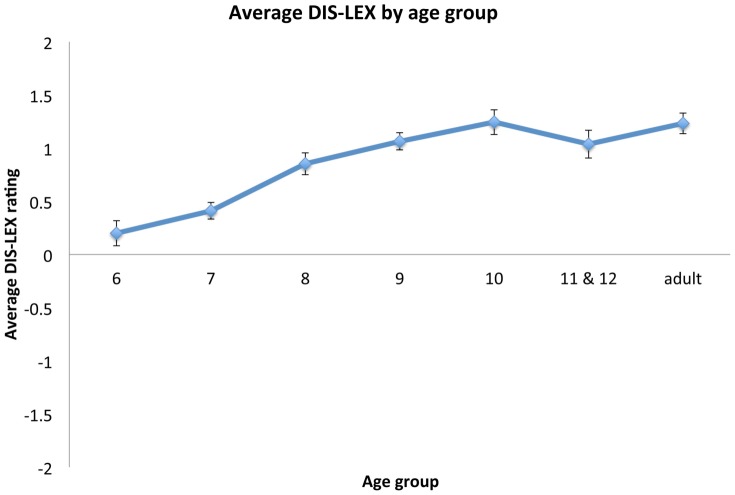
Average DIS-LEX by age group. There was a significant developmental shift even between 6^th^ and 8^th^ grades in the difference between the ratings of DIS items and the ratings of LEX items.

There was a significant effect of grade on DIS-LEX as well (*F*(7, 281) = 27.02, *p*<.001). Post-hoc Tukey HSDs revealed a several differences between age groups, most notably a significant increase even between sixth grade (*M* = .34, SD = .43) and eighth grade (*M* = .69, SD = .49) (*p* = .002), and further increases into high school and adulthood (see Figure 1). Students in tenth grade and above did not differ significantly from adults (*p*s>.9), and ninth graders differed only marginally significantly from adults (*p* = .051), suggesting that adult-like performance is reached around tenth grade. However, it is worth noting that even sixth graders tended to see DIS items as more relevant than LEX items (*t*-test against 0, *t*(50) = 5.63, *p*<.001).

Independent analyses of DIS and LEX revealed that there was a significant effect of age on DIS (*F*(7, 281) = 18.67, *p*<.001), but not LEX items (*F*(7, 281) = 1.533, n.s.). This suggests that developmental changes in the perceived relevance of DIS items drive the developmental DIS-LEX effect. Post-hoc tests confirmed that high school and adult age groups rated DIS items higher than middle school age groups, and furthermore there was a significant difference between sixth graders (*M* = 1.85, SD = .062) and eighth graders (*M* = 2.18, SD = .068) (Tukey HSD, *p*<.001). In contrast, for LEX items, all age groups were near the floor rating of 1, even for the group with the highest LEX rating (sixth graders, *M* = 1.52, SD = .516).

## Discussion

We find a strong developmental shift during adolescence in evaluations of search engine results. There was a developmental effect in the ability to recognize strongly related “HIT” items over unrelated “MISS” items, which is not wholly unexpected given previous results on children's performance on internet search tasks. More importantly, a novel developmental effect was found in the ability to recognize deeper discipline-based relationships in the absence of lexical similarity. The ability to recognize the importance of this deeper, conceptual relationship seems to increase steadily into the high school years and hits ceiling around tenth grade. However, our results also show that even sixth graders did not rely on lexical similarity alone. There was no developmental effect for items that had only surface lexical similarity to the search query in this study. Every age group accurately recognized that lexical similarity in the absence of disciplinary relatedness did not indicate relevance. This suggests that younger adolescents have specific difficulty recognizing deeper discipline relationships in isolation. Discipline relationships must be supported by other information in order for adolescents to recognize them, which extends with the findings of Iwata et al. (2011). Future studies might focus on training interventions that would allow younger adolescents to identify deeper discipline relationships more readily, such as ensuring students have better contextual understanding of the search task [7].

There is good news in these results. Adolescents were not easily misled in their search for knowledge. Rather, they have trouble recognizing all the information available to them. Given that adolescent usage of internet search engines is expanding throughout the world and that many searches are about topics with real consequences, such as personal health [14], it is critical that they do not falsely find relevance in surface lexical similarity. However, it is also important that they are able to recognize relevant information, even when it is not entirely obvious.

There are many limitations in the methods employed here. By using only simulated search results and artificially manipulating two abstract qualities, we cannot claim that our stimuli perfectly match what adolescents would encounter in the real world. However, this is a common limitation in studies of search-result evaluation, simply because real-world search results are rarely controlled for the manipulations of interest. Furthermore, while the schools that participated in this study all had computer labs available to their students and encouraged the use of the internet in educational activities, we had no explicit measure of individual participants' familiarity with internet searches. Therefore, further work would be needed to make broader generalizations about these findings in the context of individual differences in day-to-day internet use. Despite these limitations, these findings do demonstrate an important shortcoming in young adolescents' ability to identify relevant information online, despite their “digital native” status.

This study also adds a new insight into to the role of discipline-based relatedness. In line with previous findings, our results suggest difficulty with discipline-based relatedness in the absence of lexical similarity as late as eighth grade. Although previous studies have indicated that discipline-based clustering is difficult as late as sixth grade [10], this is the first indication of difficulty for older children. Furthermore, this study shows the importance of the ability to cluster knowledge based on discipline in a real-world context.

There is ample ground for future work based on these findings. The first is simply to ask whether our findings indicate that middle school students will not use discipline-based clustering by default, as much younger children are at least capable of it [11]. Our findings cannot distinguish if adolescents generally default to surface cues or if this effect is limited to the context of internet searches, where previous work suggests that they place great trust in the search engine to find the best information [8].

A second question asks why difficulty with discipline-based clustering persists through middle school. It is possible that younger children simply lack the factual knowledge to identify two things as being disciplinarily related, which would depend on the specific stimuli. For example, if a child did not know that electrical resistance causes emission at various wavelengths depending on the material, they might not realize than information about the resistance of certain metals is relevant to the understanding of how lightbulbs function. Indeed, our results could be explained in those terms, but in that case why should age groups that gave low ratings to DIS items still succeed at identifying HIT items? In theory, if they lack the knowledge to understand the disciplinary relatedness in the HIT items, then these items should be no more obviously relevant than LEX items. Rather, it is more likely that they have the knowledge to identify disciplinary relationships but are unable to do so without other cues. Future studies could tease this apart further by evaluating students' knowledge of each topic independently of their ability to recognize disciplinary relatedness.

Previous work also suggests that younger students fail to recognize the importance of disciplinary relatedness over and above other forms of relatedness. What fosters the emergence that recognition in late childhood? Perhaps it is driven by older children's confidence in their ability to create discipline-based knowledge clusters of their own as their knowledge increases, or perhaps greater experience with unreliable surface cues and their drawbacks. Conceptual development is clearly needed that goes beyond simply having extensive search experience, which is reported to be commonplace even among the youngest age group we examined. Insights into these mechanisms might well support new strategies for helping a younger generation of digital natives be more sophisticated in their searches.
